# Generalized genetic liability to substance use disorders

**DOI:** 10.1172/JCI172881

**Published:** 2024-06-03

**Authors:** Alex P. Miller, Ryan Bogdan, Arpana Agrawal, Alexander S. Hatoum

**Affiliations:** 1 Department of Psychiatry and; 2 Department of Psychological and Brain Sciences, Washington University in St. Louis, St. Louis, Missouri, USA.

## Abstract

Lifetime and temporal co-occurrence of substance use disorders (SUDs) is common and compared with individual SUDs is characterized by greater severity, additional psychiatric comorbidities, and worse outcomes. Here, we review evidence for the role of generalized genetic liability to various SUDs. Coaggregation of SUDs has familial contributions, with twin studies suggesting a strong contribution of additive genetic influences undergirding use disorders for a variety of substances (including alcohol, nicotine, cannabis, and others). GWAS have documented similarly large genetic correlations between alcohol, cannabis, and opioid use disorders. Extending these findings, recent studies have identified multiple genomic loci that contribute to common risk for these SUDs and problematic tobacco use, implicating dopaminergic regulatory and neuronal development mechanisms in the pathophysiology of generalized SUD genetic liability, with certain signals demonstrating cross-species and translational validity. Overlap with genetic signals for other externalizing behaviors, while substantial, does not explain the entirety of the generalized genetic signal for SUD. Polygenic scores (PGS) derived from the generalized genetic liability to SUDs outperform PGS for individual SUDs in prediction of serious mental health and medical comorbidities. Going forward, it will be important to further elucidate the etiology of generalized SUD genetic liability by incorporating additional SUDs, evaluating clinical presentation across the lifespan, and increasing the granularity of investigation (e.g., specific transdiagnostic criteria) to ultimately improve the nosology, prevention, and treatment of SUDs.

## Introduction

Use of psychoactive substances constitutes a significant and growing international health concern. More than 4% of the global burden of disease and injury is attributable to substance use, and this burden disproportionately affects those in adolescence and young adulthood (1–3). While the health consequences of substance use are glaring, the bulk of lives lost are attributable to heavy or prolonged use of substances and subsequent development of substance use disorders (SUDs) (4–6). SUDs are characterized by not only heavy substance use, but also a constellation of symptoms that can include increased tolerance to heavy use, loss of control over use, risky use, social impairment, and physiological dependence marked by physical and psychological withdrawal following discontinuation of heavy or prolonged use. Notably, individual SUDs (e.g., alcohol use disorder, opioid use disorder) are highly comorbid, and polysubstance use is common (7–9). In the US National Epidemiological Survey of Alcohol and Related Conditions-III (NESARC-III; *n* = 36,309), individuals with one illicit SUD were 4.0 and 3.6 times more likely to have lifetime diagnoses of alcohol or nicotine use disorder, respectively (10). In addition, a prior SUD diagnosis significantly accelerates progression from use to disorder for subsequently used substances (11). Lifetime co-occurrence of SUDs is associated with a higher rate of physical and psychological comorbidity, greater severity of each individual SUD, increased psychiatric debilitation, and overall higher morbidity (12, 13). Given the high prevalence of polysubstance use among individuals with SUDs, increased likelihood of comorbid SUDs, and greater health burdens associated with multiple SUD diagnoses, it is of paramount importance that we capture the etiology of this prevailing clinical presentation.

However, co-occurrence of substance use and SUDs also presents challenges related to construct heterogeneity. Most large-scale studies evaluate lifetime co-occurrence such that multiple SUDs may occur in a temporally constrained time frame (i.e., within the same year) (7) or sequentially and even years apart (14). The self-reported timing of individual criteria or their clustering can pose challenges for fine-tuning estimates of co-occurrence. On the other hand, many forms of substance use do occur concomitantly (e.g., chasing one drug with another, co-using substances by combining them in certain preparations, substituting or complementing one drug with another) (15–17). Given the variable addiction potential of substances, progression to SUD for individual substances, even when co-used with another, can vary considerably (18–20). Further, the likelihood of comorbid SUDs may fluctuate as an individual becomes more entrenched in receiving negative reinforcement from a particularly addictive substance (21, 22). Despite this prevalent pattern of comorbid substance use and SUDs, diagnostic schemas are substance specific. Yet with the exception of drug-specific withdrawal symptomatology, the criteria used to diagnose SUDs are identical. Further, the fifth edition of the *Diagnostic and Statistical Manual of Mental Disorders* (DSM-5) (23) eliminated polysubstance dependence (i.e., collective endorsement of three or more dependence criteria across substances, regardless of criteria endorsed for an individual substance) due to lack of application in research (24). Thus, comorbid SUDs are not codified as a distinct construct, even in classification systems that include specifiers for SUDs with comorbid mental health features that may be substance induced (e.g., International Classification of Diseases [ICD]) (25).

Given extensive concurrent and lifetime SUD comorbidity, there are likely common risk factors predisposing to SUDs that are shared across different substances. Identifying such etiological factors could offer some potential in discovering novel interventions that target SUD development across multiple substances. Despite the absence of formal definitions of SUD comorbidity, genetic research has, for the past three decades, focused on parsing influences that are specific to each drug from those that generalize to multiple SUDs, regardless of the temporal occurrence of the individual disorders. Twin, family, and molecular genetic studies have found evidence for shared genetic factors that influence liability to multiple SUDs (see ref. 26 for a review of this literature) and go beyond shared genetic liability to substance use (e.g., trying a drug; using it at least once; using it casually, regularly, or frequently). While this common genetic vulnerability correlates with other forms of psychopathology and behavior (e.g., externalizing disorders, major depression, executive functioning) (27–29), generalized genetic liability to SUDs also represents unique covariance specific to the relationships between SUDs themselves. Importantly, given estimates suggesting that drug mechanisms with genetic support may be twice as likely to result in efficacious pharmaceutical interventions, synthesizing knowledge of the shared genetic architecture across different SUDs may offer translational insight for future treatments (30). In this Review, we outline evidence for this generalized or common genetic vulnerability to SUDs (SUD-*g*) from family, twin, and genomic studies; characterize it with respect to other behavioral phenotypes, parse it quantitatively and qualitatively from drug-specific genetic influences; and outline the possible translational validation and clinical utility of this generalized genetic liability.

## Twin and family studies

**Figure 1:**
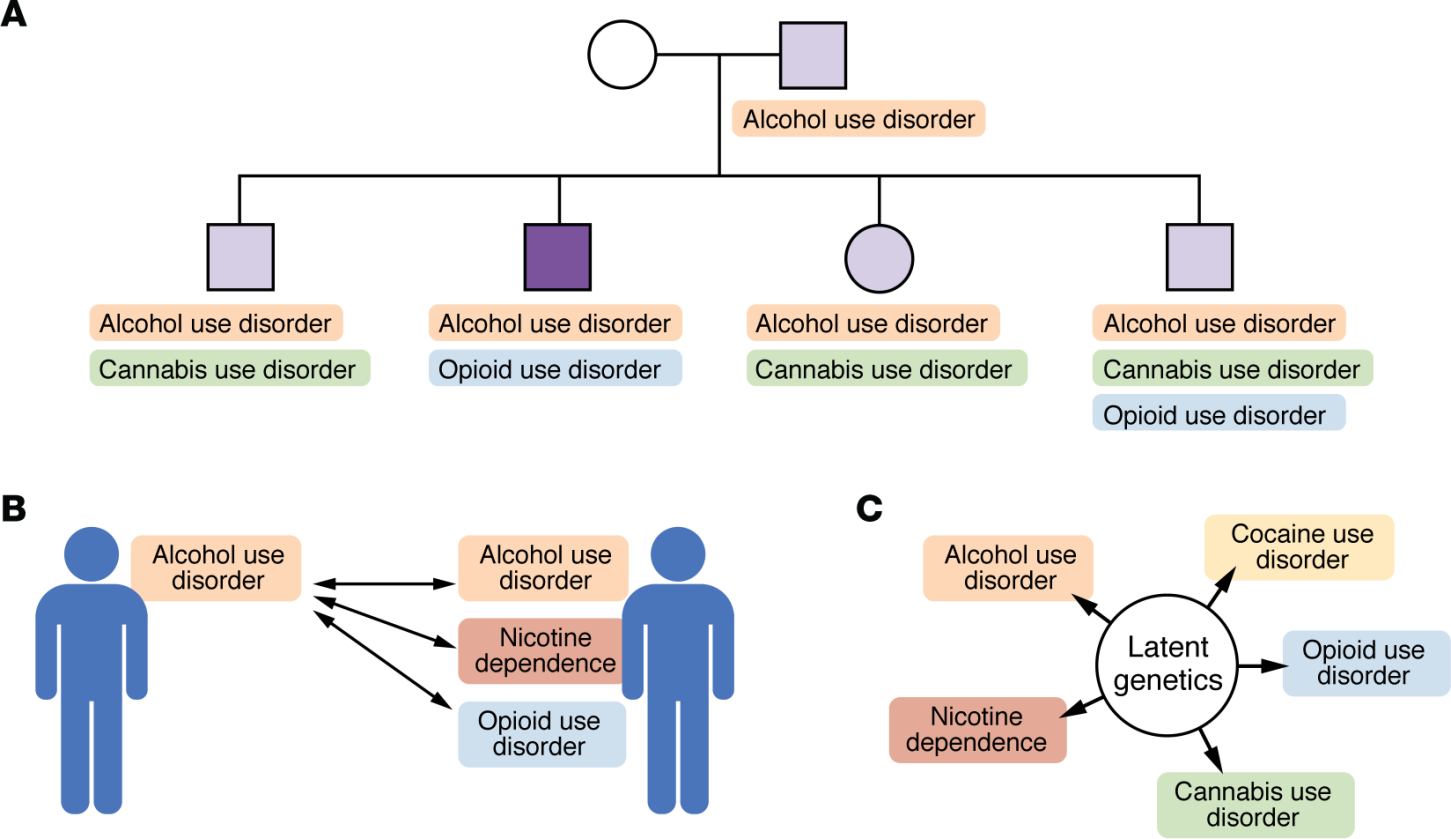
Early evidence for a generalized genetic liability to SUDs (SUD-*g*) arose from genetic epidemiological studies examining the coaggregation of various SUDs in related individuals. (**A**) Family studies found that relatives of a proband (dark purple; i.e., an index individual with a SUD) were at heightened risk for multiple SUDs. The degree of coaggregation varied by the degree of genetic relatedness; i.e., first-degree relatives such as siblings were most likely to have other SUDs. (**B**) Within twin pairs, including genetically identical monozygotic twins, there was evidence for cross-substance genetic correlations (e.g., one twin’s alcohol use disorder was associated with the other twin’s alcohol, tobacco, and opioid use disorders). (**C**) These family studies and across-SUD twin correlations led to the identification of a latent genetic factor underlying these SUDs.

Decades of family and twin research have demonstrated that SUDs are characterized by a significant heritable component, a fair proportion of which is shared across SUDs (Figure 1, A and B) (26). While only able to parse the contributions of genetic and nongenetic sources of familial similarity to a limited degree, family studies have consistently observed coaggregation of multiple SUDs in family members of probands (i.e., the individuals whose disease status identifies the family). For instance, in the Collaborative Study on the Genetics of Alcoholism, siblings of probands with alcohol dependence are considerably more likely to also meet criteria for cannabis and cocaine dependence, alongside their heightened risk for alcohol dependence itself (31) (see also ref. 32). Given the observed patterns of familial aggregation, it was anticipated that most genetic sources of liability to SUDs would be correlated, as would a significant proportion of environmental factors.

Twin studies, especially those including monozygotic (i.e., identical) and dizygotic (i.e., fraternal) twins — under the assumption of “equal environments” (i.e., some nongenetic sources of variance are shared to the same degree, regardless of zygosity [ref. 33]) — can parse genetic and nongenetic sources of familial coaggregation. Collectively, these foundational twin studies have established that the heritability of SUDs generally ranges between 30% and 80%, with a consensus estimate of 50% (26). Beyond providing estimates of heritability, twin studies have also served to test models that implicate both substance-specific and cross-cutting common vulnerability to SUDs (29, 34–39) and emphasize potentially important distinctions in coheritability between substance use, heavy use, and subsequent SUDs (40–44). Substance use is heritable to a somewhat lesser degree than SUDs and is, during adolescence, also influenced by latent environmental influences that are shared by members of twin pairs (i.e., “common” or “familial” environment) (45, 46). Consistent with this, bivariate and multivariate associations between substance use phenotypes are partially attributable to this common environment and less so to the shared genetic liability that plays such a robust role in SUD comorbidity (e.g., refs. 47, 48). This distinction in the relative contribution of shared genetic liability between substance use and SUDs exists despite the observation of fairly high latent genetic correlations between phenotypes representing earlier stages of substance use and later SUDs (44, 49–52).

Support for substantial shared genetic liability, and limited substance-specific residual genetic variance, for SUDs (previously termed substance abuse or dependence) involving “licit” (typically alcohol and nicotine) and “illicit” substances (typically cannabis, cocaine, hallucinogens, and nonprescription sedatives and stimulants) arises from multiple independent twin studies (34–39, 44, 53). Notably, these studies provided support for nicotine-specific genetic influences but not for specific genetic influences on other SUDs (e.g., refs. 37 and 47). Concurrent and subsequent work has suggested that shared genetic liability to SUDs may be characterized along with other externalizing disorders (i.e., antisocial personality disorder, conduct disorder) to reflect a broad externalizing liability (54–57), though evidence of shared liability between internalizing features and SUDs also exists (29, 58). Finally, twin studies have also served to highlight developmental differences in the relative contributions of environmental and genetic influences on substance use as well as SUD onset, progression, and co-occurrence (53, 59–62). For heritability of both substance use (59, 63) and SUDs (53, 64), the role of shared latent genetic influences may fluctuate across development, with some evidence that genetic factors have greater influence on generalized risk earlier in life and nonshared, environmental, substance-specific factors take on potentially increasing importance in adulthood (53). Together, prior family and twin studies have paved a foundational path for contemporary GWAS seeking to elucidate the complex genetic architecture of SUDs and identify molecular mechanisms and associated SNPs that confer generalized risk for SUD development (65).

## GWAS of individual SUDs demonstrate shared genetic liability

Many initial SUD GWAS were conducted using single-cohort designs and thus were relatively underpowered. Even with meta-analyses across samples, findings for cannabis, cocaine, and opioid use disorders were scant and irreproducible (26). Surprisingly, while twin studies suggested substantial shared genetic liability across multiple SUDs, the first GWAS of alcohol and nicotine phenotypes, including their problematic use, identified missense variants in genes that encode substance-specific metabolizing enzymes or neurotransmitter mechanisms (e.g., rs1229984 in *ADH1B* [encoding alcohol dehydrogenase 1B] for alcohol dependence; rs16969968 in *CHRNA5* [encoding the α5 subunit of a nicotinic acetylcholine receptor] for nicotine phenotypes) (66–69). As SUD GWAS sample sizes began to increase, replicable evidence of genome-wide significant associations began to emerge (70–72), extending the scope of discovery beyond *ADH1B* and *CHRNA5*. These larger GWAS permitted the estimation of genomic correlations underlying multiple SUDs that substantiated findings of a general SUD liability from the family and twin literature (73–75).

The first large-scale meta-analytic GWAS of SUDs focused on alcohol dependence (76). This effort was followed by a large-scale GWAS of ICD-coded alcohol use disorder (77) and a problematic alcohol use GWAS meta-analysis combining the alcohol dependence and alcohol use disorder GWAS as well as a GWAS of a questionnaire-based assessment of problem drinking (78). At the same time, large-scale GWAS of substance-use phenotypes (e.g., typical number of alcoholic drinks consumed per week, typical number of cigarettes smoked per day, lifetime cannabis use) (79, 80) began to facilitate genetic correlation (SNP-*r_g_*) analyses examining unique and shared genetic influences on use of substances and SUD diagnoses. Collectively, these studies demonstrated that genome-wide SNP contributions shared among substance-use phenotypes and between use and use disorder of the same substance (e.g., drinks per week and alcohol dependence/alcohol use disorder) are substantial (SNP-*r_g_* = 0.48–0.78) but significantly different from 1, corroborating prior conclusions from twin research. Genetic correlation analyses of these GWAS have also consistently revealed SNP contributions to alcohol dependence/alcohol use disorder that are partially distinct from contributions to alcohol use (i.e., drinks per week), with alcohol use disorder having higher genetic correlations with psychopathology and drinks per week having higher associations with anthropometric traits (77).

These findings have been replicated across large-scale GWAS meta-analyses of other SUDs (81, 82). For example, while the estimated genetic correlation between lifetime cannabis use and cannabis use disorder is high (SNP-*r_g_* = 0.50), prior and recent GWAS meta-analyses indicate marked differences in genetic correlations between other traits and cannabis use versus use disorder (81, 82). In a recent GWAS of cannabis use disorder, Levey et al. (82) found that while both cannabis use and cannabis use disorder were genetically correlated with increased neighborhood deprivation, cannabis use disorder was correlated with lower educational attainment, whereas cannabis use was associated with higher educational attainment. In aggregate, these GWAS findings have reemphasized important differences in the genetic influences on substance use versus SUDs and suggest that substance use may be of limited utility as a direct genetic proxy for SUDs.

Until recently, a considerable impediment to comprehensively estimating genetic commonality among SUDs was the lack of well-powered GWAS of SUDs other than alcohol dependence/alcohol use disorder. As large-scale meta-analytic GWAS initiatives have extended to other SUDs, greater resolution of the degree of common liability among SUDs has emerged. For instance, in the most recent GWAS of tobacco use disorder by Toikumo and colleagues (83), genetic correlations with problematic alcohol use (SNP-*r_g_* = 0.61), cannabis use disorder (SNP-*r_g_* = 0.64), and opioid use disorder (SNP-*r_g_* = 0.47) were indicative of shared genetic liability. Similarly, recent GWAS meta-analyses have demonstrated even greater genetic correlations between opioid use disorder and alcohol use disorder (SNP-*r_g_* = 0.68–0.70) and opioid use disorder and cannabis use disorder (SNP-*r_g_* = 0.65–0.82) (84, 85). In fact, genetic correlations between certain SUDs (e.g., cannabis use disorder and opioid use disorder) remain some of the highest among psychiatric disorders and are comparable to genetic correlations between schizophrenia and bipolar disorder (SNP-*r_g_* = 0.68) (86) and between major depressive disorder and generalized anxiety disorder (SNP-*r_g_* = 0.72) (87).Thus, the emerging consensus from contemporary large-scale GWAS efforts has supported a high level of shared genetic architecture of common variants underlying multiple SUDs that are distinct from genetic contributions to substance use.

## Identifying loci influencing generalized genetic liability to SUDs

**Figure 2:**
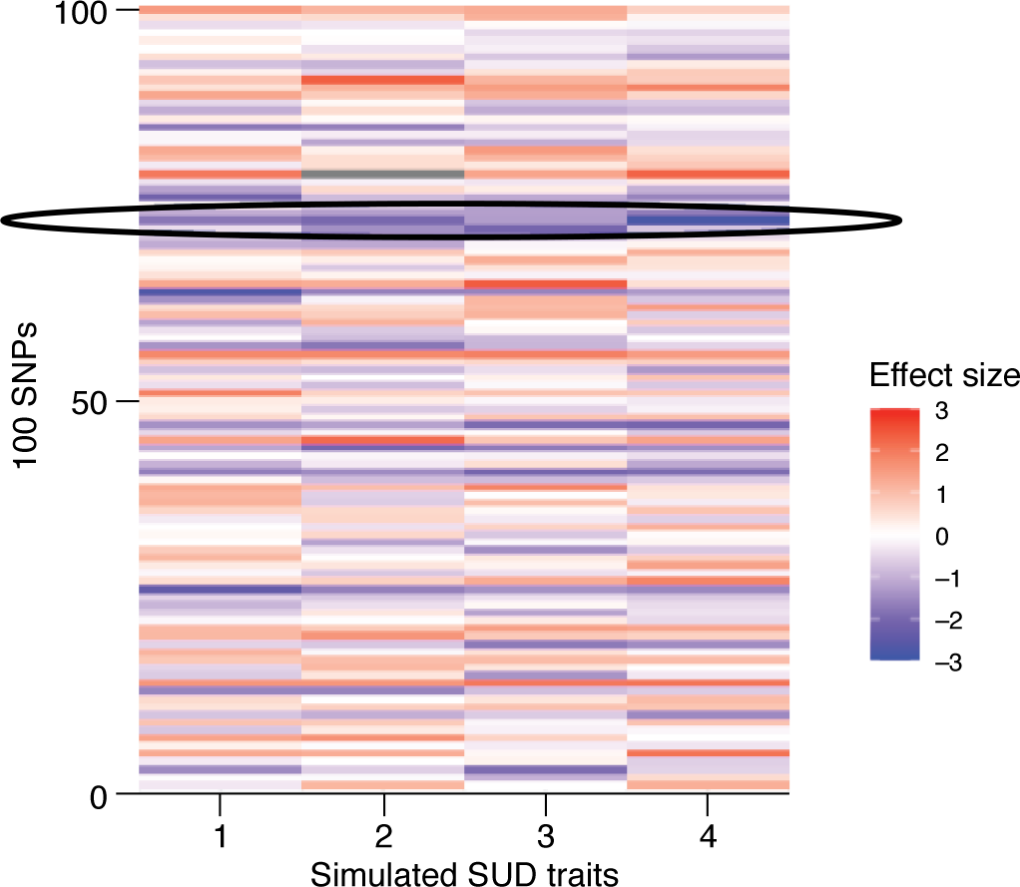
Illustration of common SNP effect sizes that contribute to underlying latent factor of genetic risk. Simulated effects of 100 independent SNPs predicting four simulated SUD traits are plotted as a heatmap. A genetic correlation of 0.70 is assumed between all traits, consistent with genetic correlations between SUDs. When a SNP effect is more similar across all four traits, that SNP will have a larger contribution to the latent underlying genetic predisposition. A single row is circled to demonstrate such consistency across the effect sizes of a SNP on the four traits. As the risk allele for the SNP in the circled row has a similar magnitude of negative effect on all four traits, that SNP is likely contributing to the inheritance of each trait and is likely a “common” effect. Many rows follow a similar consistent pattern of effect sizes, while others do not. This highlights that a genetic correlation of 0.70 includes both SNPs that capture consistent, or shared, effects, but also SNPs that may have specific effects on each trait.

Family and twin studies, as well as genetic correlations from GWAS, suggest that loci undergirding individual SUDs are likely to be largely overlapping alongside important substance-specific risk. However, the variants, genes, and pathways constituting this coheritability were yet to be identified. GWAS of individual SUDs were highly correlated, hinting at a degree of molecular similarity, but no study had identified the variants that constituted this genetic commonality. Further, given that genetic correlations across SUDs were among the largest observed for psychiatric disorders, it was hypothesized that modeling overlap across SUDs would increase sample size and discovery power in the GWAS by leveraging this genetic similarity. Figure 2 provides an illustration of how SNPs with similar effects on four simulated SUD traits might influence the genetic correlations across these traits.

**Figure 3:**
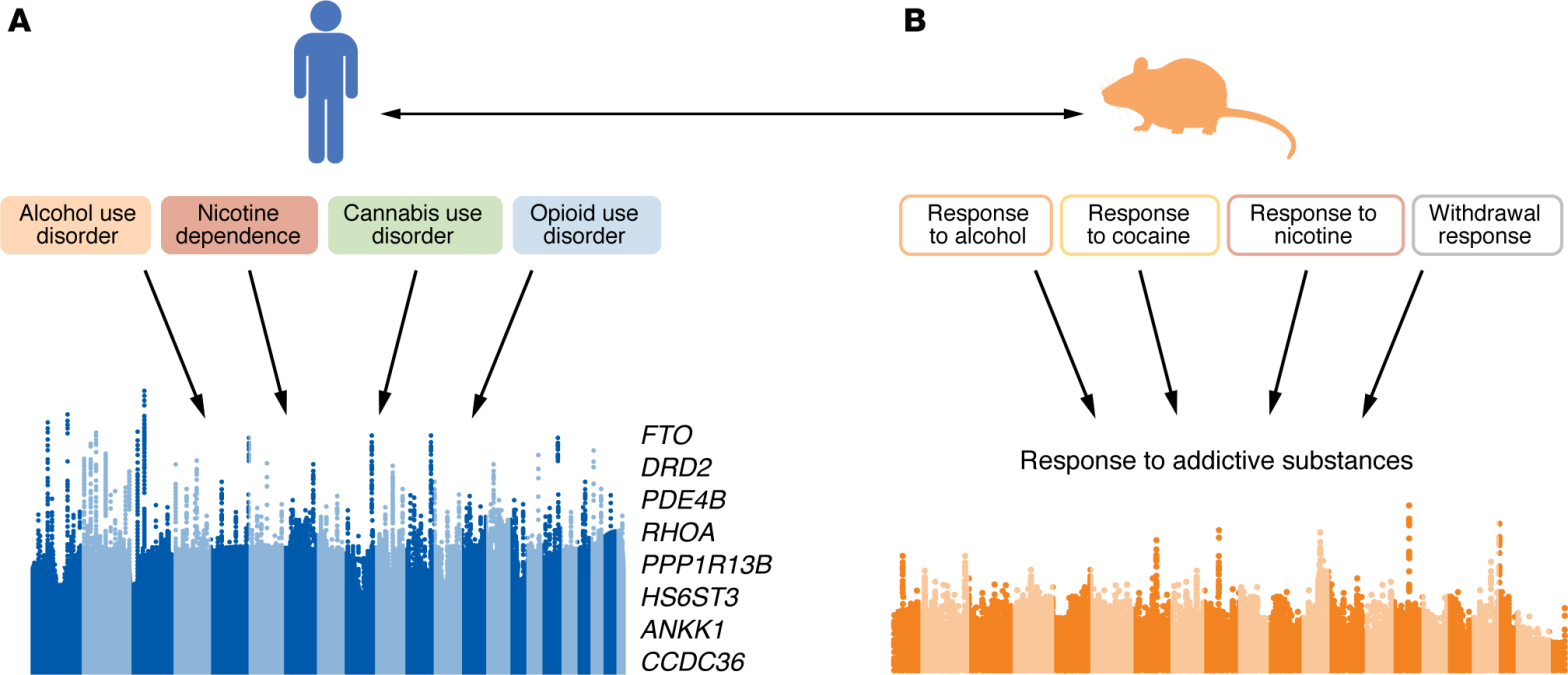
Leveraging genome-wide data to identify pleiotropic effects of common genetic variants on liability to multiple SUDs. (**A**) Large GWAS of SUDs led to the identification of the loci that shaped SUD-*g*, with loci implicated in the correlated genetic architecture. The top eight gene-based findings are listed. Representative data was redrawn based on a figure in (89) with permission of Springer Nature Limited, which retains rights to the reference image. (**B**) Corroboration of several genes implicated in the human GWAS also arises from recent meta-analyses of 5 mouse traits indexing a similar SUD-*g* response (94).

Based on this concept, in an initial study our group (Hatoum et al. 2022; ref. 88) hypothesized that a single common genetic factor would adequately explain the GWAS-derived genomic architecture underlying alcohol, tobacco, cannabis, and opioid use disorders (Figure 3A). Fitting data from the then largest GWAS of cannabis use disorder, opioid use disorder, problematic alcohol use, and problematic tobacco use to this model provided substantial support for the hypothesis that shared genomic influences contribute to genetic risk for each individual SUD. Beyond supporting this hypothesis, some additional observations arose from the initial Hatoum et al. study. First, the common genomic factor (referred to as “addiction risk factor” in that publication and herein as “generalized genetic liability to SUDs” and “SUD-*g*”) provided good fit to these data even when GWAS of substance use (i.e., drinks per week, cannabis use, and tobacco use) were controlled for, suggesting that generalized genetic liability to SUDs is partially independent of genomic influences on substance use. Interestingly, a similar confirmatory single-factor model that also included substance use phenotypes did not fit the data well, possibly due to low genetic correlations between tobacco dependence and drinks per week. Second, SUD-*g* was genetically correlated with behavioral traits associated with the development of SUDs, including executive functioning, neuroticism/negative emotionality, and risk taking, and with other psychiatric disorders, including schizophrenia, major depressive disorder, and attention-deficit/hyperactivity disorder. Yet a linear combination of substance use phenotypes, behavioral correlates of SUDs, and other psychiatric disorders did not fully explain SUD-*g*. Taken together, the observations reported by Hatoum et al. pointed to a psychometrically valid construct representing genetic liability to multiple substance use problems and disorders that is distinct from the genetics of substance use, common behavioral correlates, and general psychopathology.

This genetic commonality hypothesis can be extended to a framework for discovery of loci by including the degree of SNP effects on each between-SUD genetic correlation in the model, improving the power to discover loci by leveraging the similarity in patterns of SNP effects across SUDs. This approach led to one of the largest (~1 million individuals) multivariate GWAS of SUDs to date (Hatoum et al. 2023; ref. 89) (Figure 3A). Seventeen independent genomic loci were identified in this study as significant contributors to SUD-*g*, and pathway analysis of gene-based results implicated genes that regulate nervous system and synapse development. Further, specific genes identified through gene-based analyses have been shown to be involved in upstream regulatory processes of the neurotransmitter dopamine (e.g., *DRD2*, *PDE4B*, *BDNF*, and *FTO*), though *PDE4B* and *FTO* likely influence more-general processes not necessarily specific to dopamine. Modeling SUD-*g* also led to the isolation of loci with substance-specific effects. As with prior twin models, the expectation was that variants related to specific metabolic pathways or neurotransmitter and drug-response mechanisms associated with individual drug classes would arise as substance-specific genetic effects. Consistent with this, many of the well-characterized variants that influence substance metabolism or binding, such as *CHRNA5* and *CHRNB2* for tobacco, *ADH1B* for alcohol, and the μ opioid receptor *OPRM1* for opioids, acted at the substance-specific level. Some of these substance-specific effects replicate the largest associations observed in individual SUD GWAS (e.g., *ADH1B* for alcohol use disorder), suggesting that this residual genetic variance is also a reliable source of genetic vulnerability to SUDs.

The lower factor loading compared with other SUDs for nicotine- and tobacco-related phenotypes in SUD-*g* and prior twin analyses may have arisen due to the use of a different metric for measuring problematic tobacco use. While SUDs were typically evaluated using diagnostic classification schemes that included an assessment of psychiatric burden of SUDs, such as the ICD or the DSM, problematic tobacco use was assessed using GWAS of cigarettes per day and the Fagerström Test for Nicotine Dependence (SNP-*r_g_* = 0.97) (88), both of which, in part or whole, assess level of use. Moreover, while an excellent index of physiological dependence, the Fagerström test is only weakly correlated with other measurement schemes (90, 91). It is also possible that nicotine dependence reflects not only a genetic propensity for nicotine use, but also aero-respiratory and other physiological adaptations to combustible carcinogens common to cigarettes, the prevalent form of nicotine use in most GWAS cohorts. Prior studies have also found that variants with specific effects on nicotine dependence have opposing effects on cocaine dependence, potentially reflecting more nuanced cross-drug differences (92, 93).

## Cross-species support for generalized genetic liability to SUDs

Many mouse strains show differential patterns of substance preference and response that can be used to model SUDs (e.g., conditioned place preference, intravenous substance self-administration) (94, 95), and recent animal research has also corroborated SUD-*g* findings. A GWAS meta-analysis in mouse model data — aggregating genomic variation across all mouse laboratory strains and across many SUD-related laboratory behavioral paradigms (e.g., “anxiety-depression withdrawal response to substances,” “binge-drinking”) (96), with evidence supporting the efficacy of inhibiting the cAMP-hydrolyzing enzyme phosphodiesterase 4 (PDE4) — also identified *PDE4B* as a gene exerting general influence across SUD phenotypes (Figure 3B) (94). Relatedly, a mouse mutant model of *CADM2*, a gene implicated by past GWAS of impulsivity (97) and multiancestry fine-mapping of SUD-*g* (89), was tested on a large battery of behavioral tasks (“MouseWAS”) and showed poor performance in cognitive tasks, with BMI and impulsivity higher than those in wild-type mice (98). These results highlight a potential cross-species framework for evaluating some key aspects of generalized genetic liability for SUDs.

## SUD-*g* indexes the cumulative health burden of SUDs

Representing the aggregated effects of common variants across the genome and derived from GWAS summary statistics, polygenic scores (PGS) can provide estimates of individual genetic liability to specific traits or disorders, relative to a population (99). Consistent with observations that leveraging similar or related traits in multivariate GWAS analyses improves the predictive power of PGS (100), PGS derived from the SUD-*g* GWAS explained 2–3 times more variance than PGS derived from GWAS of any one individual SUD alone when examining the likelihood of SUD diagnoses in an independent sample (89). Not only were SUD-*g* PGS associated with SUDs represented in the model (i.e., alcohol, cannabis, opioid, and tobacco use disorder), but they were also associated with cocaine use disorder, validating the future generalizability of the polygenic structure of SUD-*g*.

**Figure 4:**
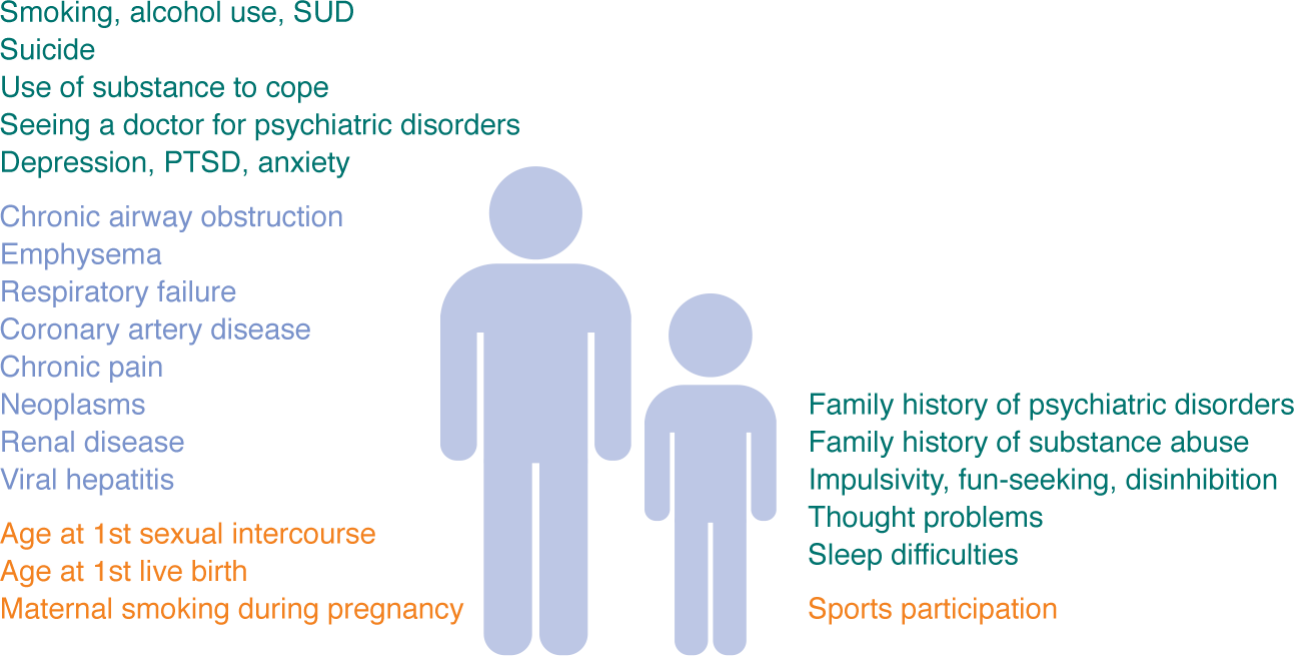
Phenome-wide associations between SUD-*g* and other medical and behavioral traits. In adults, the polygenic liability to SUD-*g* was associated with multiple mental and physical health conditions, notably other serious psychiatric disorders, and somatic problems. Even in 9- to 11-year-old children who had not previously used any substances, SUD-*g* polygenic liability was correlated with family history of psychiatric problems as well as behavioral traits. Associated traits shown are not an exhaustive listing of all phenotypic associations observed.

Perhaps more dramatic are findings from a phenome-wide association study (PheWAS) of the SUD-*g* PGS. Association tests conducted across electronic health record diagnoses in the Vanderbilt University Medical Center biobank (BioVU) sample (89) implicated serious psychiatric disorders, such as suicide-related behaviors as well as a range of other diseases and conditions — including chronic pain, viral hepatitis, respiratory illnesses, and other psychiatric disorders — as being associated with SUD-*g* PGS (Figure 4A). A second SUD-*g* PGS PheWAS provided insights into early-life correlates of generalized genetic liability to SUDs. Correlating the SUD-*g* PGS with approximately 1,400 traits in a sample of approximately 4,500 children in the Adolescent Brain Cognitive Development (ABCD) study (9–11 years of age), most of whom had not used substances (beyond sipping alcohol), uncovered associations with family history of psychiatric diagnosis, psychiatric hospitalization, and substance use problems; behaviors typically considered early markers of SUD progression, such as sensation seeking, childhood thought problems, and childhood externalizing behavior; and characteristics typically viewed as consequences of SUDs, such as number of sleep disorder symptoms/sleep duration; substance use during pregnancy and consequent prenatal exposure; and socioeconomic disadvantage (89) (Figure 4B). In particular, findings with sleep are interesting, as sleep difficulties are frequently studied as consequences of SUDs (101), while these data (and other data in adults) (102) suggest that adolescents with higher genetic liability to SUDs may have preexisting sleep challenges.

Taken together, these PheWAS of SUD-*g* have provided resounding evidence for the psychiatric burden associated with generalized genetic liability to SUDs. However, PGS still perform below thresholds of clinically meaningful prediction, and therefore, their current use in clinical settings is limited. Indeed, SUD-*g* PGS accounts for approximately 5% of variance in SUD constructs within independent samples. While this is a marked improvement upon prior PGS performance (89, 103), it is much less predictive of SUD than other well-established related risk factors such as family history (104). In adolescent populations, there are also ethical considerations to using PGS to prognosticate the likelihood of outcomes such as SUDs. The likelihood of stigma, despite efforts to destigmatize SUDs, or potential denial of medical care should be weighed against the potential for early knowledge of polygenic risk to help tailor preventative efforts and interventions, especially when PGS become predictive at clinically meaningful levels (105). However, like propensity scores used in epidemiological and clinical research to match individuals on key characteristics, a PGS can be used to characterize individuals at various levels of genetic susceptibility. When used in conjunction with data on other factors known to influence disease progression (e.g., family history, lifestyle, comorbidities) (106), PGS could facilitate patient stratification for research purposes and participation in clinical trials (e.g., ref. 107).

## Potential of SUD-*g* for drug repurposing

Recent work by our group has shown that increased sample sizes can improve the reliability of GWAS discovery of psychopharmaceuticals (108). Notably, SUD-*g* signals were enriched for gene targets for current SUD medications, suggesting that other genes implicated by the SUD-*g* GWAS may also be useful in identifying repurposable medications for treating SUDs. These potentially novel medication targets include *PDE4B*, which is targeted by drugs such as ibudilast and has shown promise in multiple clinical trials of different SUDs (109, 110); dopamine agonists, such as talipexole and lisuride, which are used in the treatment of Parkinson’s disease; the serotonin and dopamine agonist terguride and selective norepinephrine reuptake inhibitors, such as reboxetine; and varenicline, a drug already approved by regulatory agencies for the treatment of tobacco use disorder. While these medications are promising, clinical trials are needed to validate them in the treatment of individual SUDs and SUD comorbidity. Nevertheless, there is compelling potential for GWAS-fueled discoveries to generate additional medication targets as SUD-*g* sample sizes increase.

## Alternative conceptualizations of substance-related genetic liability

Several other multivariate GWAS models have examined the shared genetic liability underlying multiple substance use or SUD phenotypes, although none aside from SUD-*g* have focused on modeling SUDs exclusively. For instance, a study modeling a single common genetic liability factor underlying tobacco use frequency (cigarettes per day), tobacco use disorder, cannabis use frequency, cannabis use disorder, alcohol use frequency (drinks per week), and alcohol use disorder identified a proportion of the same loci as those arising from SUD-*g* (111). However, jointly modeling both substance use and SUD phenotypes resulted in the characterization of putative substance-specific loci (e.g., *ADH1B*, *KANSL1*, and *KLB*) as exerting generalized effects on multiple substances. Relatedly, the common genetic factor underlying these substance use and SUD phenotypes was genetically correlated with phenotypes found in previous studies to relate only to substance use rather than SUD (e.g., BMI) (89, 111).

Another study modeled the genetic liability shared by substance use (i.e., lifetime cannabis use, lifetime smoking initiation) and problematic alcohol use, along with indices of behaviors potentially representing low self-regulation, including number of sexual partners, age at first sexual intercourse, general risk tolerance, and attention-deficit/hyperactivity disorder (112). However, there are noteworthy distinctions between the traits collectively referred to as externalizing traits in this GWAS common factor model and the traits and disorders that have typified the externalizing spectrum in the literature (113, 114). Specifically, GWAS of common externalizing traits and disorders such as antisocial behavior (115, 116), conduct and oppositional defiant disorder (117), and trait aggression (118, 119) were not included in this genetic model (largely due to their smaller sample sizes) but demonstrate moderate to high genetic correlations with substance use, SUDs, and other externalizing traits (115, 117, 119, 120).

Consistent with evidence from twin studies demonstrating shared genetic influences between other externalizing disorders (e.g., antisocial personality disorder, conduct disorder) and SUDs (29), there is a notable genetic correlation between the externalizing and SUD-*g* GWAS summary statistics (SNP-*r_g_* = 0.63) (89). This finding highlights that there is considerable overlap and commonality of genes across a spectrum of potentially normative and problematic externalizing behaviors to psychiatric disorders, including SUDs (121). However, while twin studies have typically focused on maladaptive externalizing traits and disorders, the externalizing GWAS utilized both maladaptive and potentially adaptive aspects of externalizing. Indeed, comparisons of genetic correlations with other traits suggest that this externalizing genetic factor is more strongly associated with substance use and impulsivity phenotypes, while SUD-*g* is more strongly associated with psychopathology and medical morbidities (89, 112, 121). For example, drug experimentation demonstrates one of the highest genetic correlation estimates with the externalizing genetic factor (SNP-*r_g_* = 0.91) (112). In contrast, SUD-*g* correlates strongly with psychiatric disorders such as major depressive disorder (SNP-*r_g_* = 0.56) and schizophrenia (SNP-*r_g_* = 0.46) and self-medication phenotypes (“Substances taken for anxiety: Drugs or alcohol [more than once]”; SNP-*r_g_* = 0.64; refs. 89, 121). These findings suggest that prioritizing phenotypes that more specifically index SUDs in genetic discovery efforts for generalized genetic liability to SUDs may lead to better approximation of patterns of morbidity seen in epidemiological studies of lifetime and comorbid SUDs, which while co-occurring with other aspects of self-regulation are also phenotypically and genetically distinct from them.

There are also important clinical distinctions between models that include metrics of self-regulation or substance consumption, such as the externalizing GWAS, and models focused on generalized risk across SUDs more specifically, such as SUD-*g*. SUDs exert known clinical burden and thus correlate with various psychiatric and somatic disorders. On the other hand, many aspects of “risk-taking” or lack of self-regulatory capacity — such as number of sexual partners, impulsive personality traits, and sensation seeking — may in some socioenvironmental contexts occur within a range of normative exploratory behaviors that may even be evolutionarily adaptive to some degree (122, 123). Similarly, alcohol consumption frequency and “casual” cannabis use (especially as legalization generates greater permissiveness) may reflect lifestyle factors associated with higher educational attainment that serve as socioeconomic resilience against future SUD development (80, 124). Thus, SUD-*g*, normative substance use, and other externalizing and self-regulation indices are theoretically correlated constructs, but their genetic risk will likely be separable across stages of SUD development and for the contexts in which substance use occurs.

## Future scientific advancements in studying SUD-*g*

While there are several future extensions that could further inform the study of generalized genetic liability to SUDs, such as placing SUD-*g* within the context of broader frameworks of psychopathology (e.g., refs. 125–127), here we focus on potential next steps for SUD-*g* more specifically. First, as larger GWAS of other SUDs and problematic patterns of substance use arise (e.g., stimulant or cocaine use disorder), genetic models may be expanded to encompass other shared and substance-specific signals, thus serving as a framework for discoveries beyond individual SUD GWAS. A related potential future direction includes incorporating other “addiction-like” behaviors, such as pathological gambling, into this shared genetic framework. Second, and relatedly, as GWAS sample sizes and available sources of phenotypic data grow, deep phenotyping, alternative phenotype definitions, and use of secondary or intermediate phenotypes could be leveraged to ask intriguing and nuanced questions regarding the nature of SUD-*g* (128–130). One potential approach may involve using increasingly rigorous case-control designs (e.g., specific diagnostic thresholds to define cases and use of substance-exposed versus substance-naive controls) (103, 131) to provide stronger contrasts between genetic influences on normative versus problematic substance use. Some SUD GWAS including control populations that are predominately substance naive have demonstrated strong genetic correlations with initiation of that substance (e.g., tobacco use disorder–smoking initiation, SNP-*r_g_* = 0.81) (83). Similarly, other work has demonstrated appreciable differences in GWAS and PGS findings based on the study of exposed versus unexposed control populations (131), though genetic correlations between the phenotypes these studies defined tended to be high (132). Inclusion of substance-naive control populations may provide support for a common factor model by capturing genetic signals for initiation as well as problematic use; thus, control definitions warrant further attention in future research. Similarly, criterion- or item-level data may be used to home in on especially severe transdiagnostic SUD symptoms (e.g., withdrawal) (133, 134) or provide continuous indices of SUD severity (e.g., symptom counts) (133). Finally, secondary SUD phenotypes such as heavy consumption or progression from use to disorder may augment our understanding of genetic pathways influencing SUD development (135–137). Collectively, these phenotyping approaches may serve to further reduce SUD-*g* heterogeneity and improve the detection of genomic loci influencing specific transdiagnostic features of SUD risk.

Third, there is a pressing need to extend the study of SUD-*g* to other global populations beyond the predominant “European” ancestries (i.e., individuals whose genomes are most similar to the genomes of individuals in the 1000 Genomes Central European population data). While SUD-*g* was estimated in a smaller subset of data from individuals of “African” ancestry (i.e., individuals whose genomes were most similar to the genomes of individuals in the 1000 Genomes African Yoruba population data), the ability to identify shared loci was markedly hindered by smaller samples for individual SUD GWAS. Notably, to date, no other multivariate GWAS models of generalized genetic liability have extended analyses beyond individuals of European ancestry. In addition to increasing sample sizes, further diversity in included cohorts is essential (138–141). It is also worth considering how regional and cultural variability in substance use patterns may influence a more diverse conceptualization of generalized SUD liability (e.g., the use of drugs such as khat in certain subregions of Africa [refs. 142, 143] or betel quid and areca chewing in South Asia [ref. 144]).

Fourth, while prior twin research has regularly demonstrated developmentally relevant genetic influences on SUDs characterized by shifting, and potentially substance-specific, heritability estimates (53, 64), large-scale SUD GWAS efforts thus far have primarily been developmentally agnostic, leveraging samples predominantly focused on phenotypic measurement in middle adulthood. More recently, however, growing sample sizes for GWAS of childhood and adolescent psychiatric (e.g., internalizing problems) (145) and comorbid health traits (e.g., BMI) (146) have begun to show promise for well-powered lifespan genomic models of psychopathology. Using a genomic structural equation modeling framework similar to that employed to estimate SUD-*g*, a recent article by Thomas et al. (147) examined developmental variability in genetic effects on alcohol consumption across adolescence, early adulthood, and middle adulthood with GWAS from three longitudinal cohort studies. Results of this study provide preliminary evidence consistent with twin studies suggesting that genetic liability for alcohol consumption in adolescence may be distinct from genetic liability for alcohol consumption in later developmental periods (62). Future work aggregating substance use data across additional developmental and longitudinally informed samples may provide a means to assess the degree to which genetic liability for substance use and SUDs, both general and substance specific, may change over the lifespan.

Finally, this review focused on common genetic influences on SUDs, but earlier twin models also implicated environmental risk mechanisms in generalized liability across SUDs (38). While environmental factors exert primary effects often exceeding those of individual genetic variants or PGS, they may also work in tandem with generalized genetic liability, via gene-environment correlations or interactions (148). The importance of studying such factors in the etiology of specific and generalized liability to SUDs, while beyond the scope of this Review, cannot be overstated.

## Conclusion

Recent large-scale GWAS have begun to identify loci and characterize the polygenic architecture that shapes a generalized genetic liability to multiple SUDs. Variants contributing to SUD-*g* largely relate to synaptic regulation and have shown early promise in identifying potential novel pharmacotherapies for SUDs. Beyond genome-wide significant signals, SUD-*g* polygenic risk correlates with serious medical conditions, such as chronic pain; other SUDs; sensation-seeking and sleep difficulties in adolescents; and in adults, many conditions frequently considered to be consequences of SUDs. In addition to these shared signals were variants encoding receptors and metabolizing enzymes specific to individual SUDs. This approach of aggregating genetic liability across SUDs in practice provided a boost in statistical power to detect genetic signals, but it is also clinically representative of the natural comorbid occurrence of SUDs. Thus, pharmacotherapeutics potentially identified by such an approach may yield widespread benefit across SUDs, including instances of SUD comorbidity where concomitant withdrawal can confer relapse risk. To better capture the etiology of SUDs, future gene discovery efforts may consider phenotypes or models that represent common clinical manifestations of SUDs, even if they are not codified in diagnostic schema.

## Version 1 06/03/2024

Electronic publication

## Version 2 06/05/2024

Added image credit to figure 3

## References

[1] Degenhardt L (2018). The global burden of disease attributable to alcohol and drug use in 195 countries and territories, 1990–2016: a systematic analysis for the Global Burden of Disease Study 2016. Lancet Psychiatry.

[2] Griswold MG (2018). Alcohol use and burden for 195 countries and territories, 1990–2016: a systematic analysis for the Global Burden of Disease Study 2016. The Lancet.

[3] Degenhardt L (2016). The increasing global health priority of substance use in young people. Lancet Psychiatry.

[4] Glantz MD (2020). The epidemiology of alcohol use disorders cross-nationally: findings from the World Mental Health Surveys. Addict Behav.

[5] Whiteford HA (2013). Global burden of disease attributable to mental and substance use disorders: findings from the Global Burden of Disease Study 2010. The Lancet.

[6] Erskine HE (2015). A heavy burden on young minds: the global burden of mental and substance use disorders in children and youth. Psychol Med.

[7] Bailey AJ, McHugh RK (2023). Why do we focus on the exception and not the rule? Examining the prevalence of mono- versus polysubstance use in the general population. Addiction.

[8] Hayley AC (2017). DSM-5 cannabis use disorder, substance use and DSM-5 specific substance-use disorders: Evaluating comorbidity in a population-based sample. Eur Neuropsychopharmacol.

[9] McCabe SE (2017). Multiple DSM-5 substance use disorders: A national study of US adults. Hum Psychopharmacol.

[10] Grant BF (2016). Epidemiology of DSM-5 drug use disorder: results from the national epidemiologic survey on alcohol and related conditions–III. JAMA Psychiatry.

[11] Marel C (2019). Conditional probabilities of substance use disorders and associated risk factors: progression from first use to use disorder on alcohol, cannabis, stimulants, sedatives and opioids. Drug Alcohol Depend.

[12] Stiltner B (2023). Polysubstance addiction patterns among 7,989 individuals with cocaine use disorder. iScience.

[13] Hjemsæter AJ (2019). Mortality, cause of death and risk factors in patients with alcohol use disorder alone or poly-substance use disorders: a 19-year prospective cohort study. BMC Psychiatry.

[14] Swendsen J (2010). Mental disorders as risk factors for substance use, abuse and dependence: results from the 10-year follow-up of the National Comorbidity Survey. Addiction.

[15] Sokolovsky AW (2020). Alcohol and marijuana co-use: consequences, subjective intoxication, and the operationalization of simultaneous use. Drug Alcohol Depend.

[16] Liu Y (2018). The importance of considering polysubstance use: lessons from cocaine research. Drug Alcohol Depend.

[17] Compton WM (2021). Polysubstance use in the U.S. opioid crisis. Mol Psychiatry.

[18] Ridenour TA (2006). Different lengths of times for progressions in adolescent substance involvement. Addict Behav.

[19] Behrendt S (2009). Transitions from first substance use to substance use disorders in adolescence: is early onset associated with a rapid escalation?. Drug Alcohol Depend.

[20] Moss HB (2014). Early adolescent patterns of alcohol, cigarettes, and marijuana polysubstance use and young adult substance use outcomes in a nationally representative sample. Drug Alcohol Depend.

[21] Peters EN, Hughes JR (2010). Daily marijuana users with past alcohol problems increase alcohol consumption during marijuana abstinence. Drug Alcohol Depend.

[22] Allsop DJ (2014). Changes in cigarette and alcohol use during cannabis abstinence. Drug Alcohol Depend.

[24] Hasin DS (2013). DSM-5 criteria for substance use disorders: recommendations and rationale. Am J Psychiatry.

[25] https://www.who.int/standards/classifications/classification-of-diseases.

[26] Deak JD, Johnson EC (2021). Genetics of substance use disorders: a review. Psychol Med.

[27] Hicks BM (2012). Index of the transmissible common liability to addiction: heritability and prospective associations with substance abuse and related outcomes. Drug Alcohol Depend.

[28] Young SE (2009). Behavioral disinhibition: liability for externalizing spectrum disorders and its genetic and environmental relation to response inhibition across adolescence. J Abnorm Psychol.

[29] Kendler KS (2003). The structure of genetic and environmental risk factors for common psychiatric and substance use disorders in men and women. Arch Gen Psychiatry.

[30] Nelson MR (2015). The support of human genetic evidence for approved drug indications. Nat Genet.

[31] Bierut LJ (1998). Familial transmission of substance dependence: alcohol, marijuana, cocaine, and habitual smoking: a report from the collaborative study on the genetics of alcoholism. Arch Gen Psychiatry.

[33] Eaves L (2003). Has the “Equal Environments” assumption been tested in twin studies?. Twin Res.

[34] Kendler KS (2003). Specificity of genetic and environmental risk factors for use and abuse/dependence of cannabis, cocaine, hallucinogens, sedatives, stimulants, and opiates in male twins. Am J Psychiatry.

[35] Kendler KS (2015). A population-based Swedish twin and sibling study of cannabis, stimulant and sedative abuse in men. Drug Alcohol Depend.

[36] Tsuang MT (1998). Co-occurrence of abuse of different drugs in men: the role of drug-specific and shared vulnerabilities. Arch Gen Psychiatry.

[37] Kendler KS (2007). Specificity of genetic and environmental risk factors for symptoms of cannabis, cocaine, alcohol, caffeine, and nicotine dependence. Arch Gen Psychiatry.

[38] Palmer RHC (2012). Genetic etiology of the common liability to drug dependence: evidence of common and specific mechanisms for DSM-IV dependence symptoms. Drug Alcohol Depend.

[39] Xian H (2008). Genetic and environmental contributions to nicotine, alcohol and cannabis dependence in male twins. Addiction.

[40] Kendler KS (2000). Illicit psychoactive substance use, heavy use, abuse, and dependence in a US population-based sample of male twins. Arch Gen Psychiatry.

[41] Verweij KJH (2010). Genetic and environmental influences on cannabis use initiation and problematic use: a meta-analysis of twin studies. Addiction.

[42] Sartor CE (2010). Common genetic contributions to alcohol and cannabis use and dependence symptomatology. Alcohol Clin Exp Res.

[43] Grant JD (2009). Alcohol consumption indices of genetic risk for alcohol dependence. Biol Psychiatry.

[44] Young SE (2006). Genetic and environmental vulnerabilities underlying adolescent substance use and problem use: general or specific?. Behav Genet.

[45] Rhee SH (2003). Genetic and environmental influences on substance initiation, use, and problem use in adolescents. Arch Gen Psychiatry.

[46] Neiderhiser JM (2013). Four factors for the initiation of substance use by young adulthood: a 10-year follow-up twin and sibling study of marital conflict, monitoring, siblings, and peers. Dev Psychopathol.

[47] Richmond-Rakerd LS (2016). Age at first use and later substance use disorder: shared genetic and environmental pathways for nicotine, alcohol, and cannabis. J Abnorm Psychol.

[48] Baker JH (2012). Shared environmental contributions to substance use. Behav Genet.

[49] Kendler KS (1999). Genetic and environmental risk factors in the aetiology of illicit drug initiation and subsequent misuse in women. Br J Psychiatry.

[50] Agrawal A (2005). Illicit drug use and abuse/dependence: modeling of two-stage variables using the CCC approach. Addict Behav.

[51] Fowler T (2007). Exploring the relationship between genetic and environmental influences on initiation and progression of substance use. Addiction.

[52] Heath AC (2002). Estimating two-stage models for genetic influences on alcohol, tobacco or drug use initiation and dependence vulnerability in twin and family data. Twin Res.

[53] Vrieze SI (2012). Decline in genetic influence on the co-occurrence of alcohol, marijuana, and nicotine dependence symptoms from age 14 to 29. Am J Psychiatry.

[54] Hicks BM (2004). Family transmission and heritability of externalizing disorders: a twin-family study. Arch Gen Psychiatry.

[55] Hicks BM (2013). Genetic and environmental influences on the familial transmission of externalizing disorders in adoptive and twin offspring. JAMA Psychiatry.

[56] Vrieze SI (2013). Three mutually informative ways to understand the genetic relationships among behavioral disinhibition, alcohol use, drug use, nicotine use/dependence, and their co-occurrence: twin biometry, GCTA, and genome-wide scoring. Behav Genet.

[57] Kendler KS (2016). A Swedish population-based multivariate twin study of externalizing disorders. Behav Genet.

[58] Burcusa SL (2003). Adolescent twins discordant for major depressive disorder: shared familial liability to externalizing and other internalizing disorders. J Child Psychol Psychiatry.

[59] Kendler KS (2008). Genetic and environmental influences on alcohol, caffeine, cannabis, and nicotine use from early adolescence to middle adulthood. Arch Gen Psychiatry.

[60] Zellers SM (2022). Developmental and etiological patterns of substance use from adolescence to middle age: a longitudinal twin study. Drug Alcohol Depend.

[61] Long EC (2019). Different characteristics and heritabilities of alcohol use disorder classes: a population-based Swedish study. Alcohol Alcohol.

[62] Edwards AC, Kendler KS (2013). Alcohol consumption in men is influenced by qualitatively different genetic factors in adolescence and adulthood. Psychol Med.

[63] Waaktaar T (2018). The genetic and environmental architecture of substance use development from early adolescence into young adulthood: a longitudinal twin study of comorbidity of alcohol, tobacco and illicit drug use. Addiction.

[64] Palmer RHC (2013). Stability and change of genetic and environmental effects on the common liability to alcohol, tobacco, and cannabis DSM-IV dependence symptoms. Behav Genet.

[65] Agrawal A, Lynskey MT (2008). Are there genetic influences on addiction: evidence from family, adoption and twin studies. Addiction.

[66] Bierut LJ (2010). A genome-wide association study of alcohol dependence. Proc Natl Acad Sci.

[67] Edenberg HJ (2010). Genome-wide association study of alcohol dependence implicates a region on chromosome 11. Alcohol Clin Exp Res.

[68] Treutlein J (2009). Genome-wide association study of alcohol dependence. Arch Gen Psychiatry.

[69] Agrawal A (2011). A genome-wide association study of DSM-IV cannabis dependence. Addict Biol.

[70] Gelernter J (2014). Genome-wide association study of opioid dependence: multiple associations mapped to calcium and potassium pathways. Biol Psychiatry.

[71] Gelernter J (2014). Genome-wide association study of alcohol dependence:significant findings in African- and European-Americans including novel risk loci. Mol Psychiatry.

[72] Gelernter J (2014). Genome-wide association study of cocaine dependence and related traits: FAM53B identified as a risk gene. Mol Psychiatry.

[73] Wetherill L (2015). Association of substance dependence phenotypes in the COGA sample. Addict Biol.

[74] Drgon T (2010). Genome wide association for addiction: replicated results and comparisons of two analytic approaches. PLoS One.

[75] Uhl GR (2008). Molecular genetics of addiction and related heritable phenotypes: genome-wide association approaches identify “connectivity constellation” and drug target genes with pleiotropic effects. Ann N Y Acad Sci.

[76] Walters RK (2018). Transancestral GWAS of alcohol dependence reveals common genetic underpinnings with psychiatric disorders. Nat Neurosci.

[77] Kranzler HR (2019). Genome-wide association study of alcohol consumption and use disorder in 274,424 individuals from multiple populations. Nat Commun.

[78] Zhou H (2020). Genome-wide meta-analysis of problematic alcohol use in 435,563 individuals yields insights into biology and relationships with other traits. Nat Neurosci.

[79] Liu M (2019). Association studies of up to 1.2 million individuals yield new insights into the genetic etiology of tobacco and alcohol use. Nat Genet.

[80] Pasman JA (2018). GWAS of lifetime cannabis use reveals new risk loci, genetic overlap with psychiatric traits, and a causal influence of schizophrenia. Nat Neurosci.

[81] Johnson EC (2020). A large-scale genome-wide association study meta-analysis of cannabis use disorder. Lancet Psychiatry.

[82] Levey DF (2023). Multi-ancestry genome-wide association study of cannabis use disorder yields insight into disease biology and public health implications. Nat Genet.

[83] Toikumo S Multi-ancestry meta-analysis of tobacco use disorder identifies 461 potential risk genes and reveals associations with multiple health outcomes. Nat Hum Behav.

[84] Kember RL (2022). Cross-ancestry meta-analysis of opioid use disorder uncovers novel loci with predominant effects in brain regions associated with addiction. Nat Neurosci.

[85] Deak JD (2022). Genome-wide association study in individuals of European and African ancestry and multi-trait analysis of opioid use disorder identifies 19 independent genome-wide significant risk loci. Mol Psychiatry.

[86] Mullins N (2021). Genome-wide association study of more than 40,000 bipolar disorder cases provides new insights into the underlying biology. Nat Genet.

[87] Levey DF (2021). Bi-ancestral depression GWAS in the Million Veteran Program and meta-analysis in >1.2 million individuals highlight new therapeutic directions. Nat Neurosci.

[88] Hatoum AS (2022). The addiction risk factor: a unitary genetic vulnerability characterizes substance use disorders and their associations with common correlates. Neuropsychopharmacoly.

[89] Hatoum AS (2023). Multivariate genome-wide association meta-analysis of over 1 million subjects identifies loci underlying multiple substance use disorders. Nat Ment Health.

[90] Moolchan ET (2002). The Fagerstrom test for nicotine dependence and the diagnostic interview schedule: do they diagnose the same smokers?. Addict Behav.

[91] Agrawal A (2011). A latent class analysis of DSM-IV and Fagerström (FTND) criteria for nicotine dependence. Nicotine Tob Res.

[92] Grucza RA (2008). A risk allele for nicotine dependence in CHRNA5 is a protective allele for cocaine dependence. Biol Psychiatry.

[93] Sherva R (2010). Variation in nicotinic acetylcholine receptor genes is associated with multiple substance dependence phenotypes. Neuropsychopharmacoly.

[94] Ball RL (2024). GenomeMUSter mouse genetic variation service enables multitrait, multipopulation data integration and analysis. Genome Res.

[95] Schoenrock SA (2020). Characterization of genetically complex Collaborative Cross mouse strains that model divergent locomotor activating and reinforcing properties of cocaine. Psychopharmacology (Berl).

[96] Jimenez Chavez CL (2021). Selective inhibition of PDE4B reduces binge drinking in two C57BL/6 substrains. Int J Mol Sci.

[97] Sanchez-Roige S (2019). Genome-wide association studies of impulsive personality traits (BIS-11 and UPPS-P) and drug experimentation in up to 22,861 adult research participants identify loci in the *CACNA1I* and *CADM2* genes. J Neurosci.

[98] Sanchez-Roige S (2023). CADM2 is implicated in impulsive personality and numerous other traits by genome- and phenome-wide association studies in humans and mice. Transl Psychiatry.

[99] Torkamani A (2018). The personal and clinical utility of polygenic risk scores. Nat Rev Genet.

[100] Turley P (2018). Multi-trait analysis of genome-wide association summary statistics using MTAG. Nat Genet.

[101] Conroy DA, Arnedt JT (2014). Sleep and substance use disorders: an update. Curr Psychiatry Rep.

[102] Hatoum AS (2022). Characterisation of the genetic relationship between the domains of sleep and circadian-related behaviours with substance use phenotypes. Addict Biol.

[103] Zhou H (2020). Association of OPRM1 functional coding variant with opioid use disorder. JAMA Psychiatry.

[104] Merikangas KR (1998). Familial transmission of substance use disorders. Arch Gen Psychiatry.

[105] Hatoum AS (2021). Ancestry may confound genetic machine learning: candidate-gene prediction of opioid use disorder as an example. Drug Alcohol Depend.

[106] Barr PB (2022). Clinical, environmental, and genetic risk factors for substance use disorders: characterizing combined effects across multiple cohorts. Mol Psychiatry.

[107] Natarajan P (2017). Polygenic risk score identifies subgroup with higher burden of atherosclerosis and greater relative benefit from statin therapy in the primary prevention setting. Circulation.

[108] https://www.medrxiv.org/content/10.1101/2023.12.05.23299434v2.

[109] Burnette EM (2021). Ibudilast attenuates alcohol cue-elicited frontostriatal functional connectivity in alcohol use disorder. Alcohol Clin Exp Res.

[110] Grodin EN (2021). Ibudilast, a neuroimmune modulator, reduces heavy drinking and alcohol cue-elicited neural activation: a randomized trial. Transl Psychiatry.

[111] Schoeler T (2023). Novel biological insights into the common heritable liability to substance involvement: a multivariate genome-wide association study. Biol Psychiatry.

[112] Linnér RK (2021). Multivariate analysis of 1.5 million people identifies genetic associations with traits related to self-regulation and addiction. Nat Neurosci.

[113] Beauchaine TP, McNulty T (2013). Comorbidities and continuities as ontogenic processes: toward a developmental spectrum model of externalizing psychopathology. Dev Psychopathol.

[114] Krueger RF (2021). Validity and utility of Hierarchical Taxonomy of Psychopathology (HiTOP): II. Externalizing superspectrum. World Psychiatry.

[115] Tielbeek JJ (2022). Uncovering the genetic architecture of broad antisocial behavior through a genome-wide association study meta-analysis. Mol Psychiatry.

[116] Tielbeek JJ (2017). Genome-wide association studies of a broad spectrum of antisocial behavior. JAMA Psychiatry.

[117] Demontis D (2021). Risk variants and polygenic architecture of disruptive behavior disorders in the context of attention-deficit/hyperactivity disorder. Nat Commun.

[118] Pappa I (2016). A genome-wide approach to children’s aggressive behavior: The EAGLE consortium. Am J Med Genet B Neuropsychiatr Genet.

[119] Ip HF (2021). Genetic association study of childhood aggression across raters, instruments, and age. Transl Psychiatry.

[120] Waldman ID (2020). Testing structural models of psychopathology at the genomic level. World Psychiatry.

[121] Poore HE (2023). A multivariate approach to understanding the genetic overlap between externalizing phenotypes and substance use disorders. Addict Biol.

[122] Casey BJ (2008). The adolescent brain. Dev Rev.

[123] Romer D (2017). Beyond stereotypes of adolescent risk taking: Placing the adolescent brain in developmental context. Dev Cogn Neurosci.

[124] Zhou T (2021). Educational attainment and drinking behaviors: Mendelian randomization study in UK Biobank. Mol Psychiatry.

[125] Kotov R (2017). The hierarchical taxonomy of psychopathology (HiTOP): a dimensional alternative to traditional nosologies. J Abnorm Psychol.

[126] Grotzinger AD (2022). Genetic architecture of 11 major psychiatric disorders at biobehavioral, functional genomic and molecular genetic levels of analysis. Nat Genet.

[127] Grotzinger AD (2021). Shared genetic architecture across psychiatric disorders. Psychol Med.

[128] Waszczuk MA (2023). Dimensional and transdiagnostic phenotypes in psychiatric genome-wide association studies. Mol Psychiatry.

[129] Sanchez-Roige S, Palmer AA (2020). Emerging phenotyping strategies will advance our understanding of psychiatric genetics. Nat Neurosci.

[130] Tiego J (2023). Precision behavioral phenotyping as a strategy for uncovering the biological correlates of psychopathology. Nat Ment Health.

[131] Polimanti R (2020). Leveraging genome-wide data to investigate differences between opioid use vs. opioid dependence in 41,176 individuals from the Psychiatric Genomics Consortium. Mol Psychiatry.

[132] Gaddis N (2022). Multi-trait genome-wide association study of opioid addiction: OPRM1 and beyond. Sci Rep.

[133] Lai D (2019). Genome-wide association studies of alcohol dependence, DSM-IV criterion count and individual criteria. Genes Brain Behav.

[134] Miller AP (2023). Diagnostic criteria for identifying individuals at high risk of progression from mild or moderate to severe alcohol use disorder. JAMA Netw Open.

[135] Kember RL (2023). Genetic underpinnings of the transition from alcohol consumption to alcohol use disorder: shared and unique genetic architectures in a cross-ancestry sample. Am J Psychiatry.

[136] Xu K (2020). Genome-wide association study of smoking trajectory and meta-analysis of smoking status in 842,000 individuals. Nat Commun.

[137] Deak JD (2022). Genome-wide investigation of maximum habitual alcohol intake in US veterans in relation to alcohol consumption traits and alcohol use disorder. JAMA Netw Open.

[138] Martin AR (2022). Increasing diversity in genomics requires investment in equitable partnerships and capacity building. Nat Genet.

[139] Martin AR (2019). Clinical use of current polygenic risk scores may exacerbate health disparities. Nat Genet.

[140] Mills MC, Rahal C (2019). A scientometric review of genome-wide association studies. Commun Biol.

[141] Fatumo S, Inouye M (2023). African genomes hold the key to accurate genetic risk prediction. Nat Hum Behav.

[142] Mihretu A (2020). Exploring the concept of problematic khat use in the Gurage community, South Central Ethiopia: a qualitative study. BMJ Open.

[143] Teferra S (2011). Khat chewing in persons with severe mental illness in Ethiopia: a qualitative study exploring perspectives of patients and caregivers. Transcult Psychiatry.

[144] Gunjal S (2020). An overview on betel quid and areca nut practice and control in selected Asian and South East Asian countries. Subst Use Misuse.

[145] Jami ES (2022). Genome-wide association meta-analysis of childhood and adolescent internalizing symptoms. J Am Acad Child Adolesc Psychiatry.

[146] Couto Alves A (2019). GWAS on longitudinal growth traits reveals different genetic factors influencing infant, child, and adult BMI. Sci Adv.

[147] Thomas NS (2024). A developmentally-informative genome-wide association study of alcohol use frequency. Behav Genet.

[148] Meyers JL, Salvatore JE (2021). Genetic and social-environmental influences on substance use and disorders. Psychiatric Annals.

